# Case report: A case of sintilimab-induced recurrent diabetic ketoacidosis and thyroid dysfunction in a patient with advanced cervical carcinoma

**DOI:** 10.3389/fimmu.2024.1405856

**Published:** 2024-10-10

**Authors:** Chunliang Wang, Ye Cai, Pei Feng

**Affiliations:** ^1^ Department of Endocrinology, The Affiliated People’s Hospital of Ningbo University, Ningbo, China; ^2^ Department of Information, The Affiliated People’s Hospital of Ningbo University, Ningbo, China

**Keywords:** immune checkpoint inhibitor, immune-related endocrine event, TIDM, DKA, thyroid dysfunction

## Abstract

Immune checkpoint inhibitors (ICIs) have radically altered cancer treatment, but immune toxicities called immune-related adverse events (irAEs), particularly endocrine toxicities, such as acute-onset diabetes and thyroid dysfunction, pose challenges. Although most irAEs have mild-to-moderate severity, failure to diagnose and treat them promptly can result in life-threatening complications. This report presents the case of a 50-year-old woman who developed ICI-induced diabetes mellitus (ICI-DM) during sintilimab treatment for advanced cervical carcinoma. The patient experienced repeated episodes of diabetic ketoacidosis (DKA) and subclinical hypothyroidism. Unlike the case of patients with typical type 1 diabetes mellitus (T1DM), our patient tested negative for β cell autoantibodies and progressed rapidly. Prompt recognition and insulin treatment are crucial for helping patients overcome such crises. Eventually, sintilimab was discontinued, and chemotherapy was initiated. This case report contributes to our understanding of ICI-DM. The significance of monitoring thyroid function and blood glucose levels before initiating ICI treatment to identify irAEs early and effectively manage them are important considerations.

## 1 Introduction

The boom in immune checkpoint inhibitors (ICIs) has changed the cancer treatment landscape. In 2019, approximately 43.6% of patients with cancer in the United States received ICI therapy, of which an estimated 12% showed a positive response (1). Although ICIs have enormous potential, their success has been limited by a range of adverse drug reactions described as immune-related adverse events (irAEs) (2). Immune-related endocrine events (irEEs), including hypoadrenia (0.7%), type I diabetes mellitus (0.2–2%), hypophysitis (5.6–11%), and thyroid disorders (30%) are common types of irAEs (3). The pathogenesis of irAEs is not completely understood, including (a) T cell-mediated mechanisms: the loss of T cell tolerance; expansion of autoreactive T cells can target shared antigens of normal and tumoral tissues; the breach in peripheral tolerance due to an imbalance of Treg cells; (b) B cells and autoAbs mediated mechanisms; (c) The activation of autoreactive B and T cells produce auto-Abs; (d) the activation of the classic complement cascade mediated tissue damage; (e) hyperinflammatory status resulting from massive cytokine release; and (f) other factors such as genetics and epigenetics, the environment and the microbiota, and the underlying immune status (4). Autoantibodies are often monitored in patients with ICI-DM or thyroid dysfunction. Furthermore, with expanding indications for ICIs and improved patient survival rates, the incidence of irAEs is expected to increase accordingly. However, toxicities associated with ICI treatment are a matter of significant clinical concern. In rare cases, these toxicities can cause treatment delays, discontinuation, and life-threatening complications. Therefore, a thorough understanding of irAEs, early diagnosis, and prompt treatment are crucial. We report the case of an older woman who received sintilimab after being diagnosed with malignant cervical cancer (IV B). The patient presented with subclinical hypothyroidism approximately 9 weeks after treatment with sintilimab. At 17 weeks, the patient developed ICI-induced diabetes mellitus (ICI-DM) with symptoms of recurrent fatigue, anorexia, nausea, and vomiting without the classic symptoms of diabetes, which was misdiagnosed as a complication of malignancy and ignored, leading to recurrent diabetic ketoacidosis (DKA). Sintilimab was discontinued owing to severe DKA symptoms.

## 2 Case description

In June 2017, a 50-year-old woman was diagnosed with cervical cancer and underwent radical surgery. Histological examination revealed the presence of a high-grade neuroendocrine carcinoma. No distant metastases were observed during tumor staging. The patient achieved a complete response after 6 cycles of cisplatin and etoposide chemotherapy combined with radiotherapy. However, the patient experienced cough and expectoration without fever, gradually worsening in June 2019. The clinical stage was T4N1M1 (IVB) based on positron emission tomography-computed tomography (PET-CT) examination, and several metastases involving the lungs, adrenal glands, and lymph nodes were observed. Following chemotherapy (5 cycles of cisplatin and etoposide, and 4 cycles of nab-paclitaxel and carboplatin), anlotinib was administered, but it was ineffective in delaying the progression of the disease. A subsequent PET-CT scan in July 2021 revealed further enlargement of the tumor with multiple metastases. Sintilimab (200 mg every 3 weeks) combined with anlotinib was initiated on July 26, 2021, and the patient showed progressive clinical improvement. During these months, the patient experienced occasional nausea that was initially misdiagnosed as a complication of malignancy because the intensity of the symptoms was not considered serious.

However, 11 days after treatment with the sixth cycle of sintilimab, the patient arrived at the emergency department with anorexia, fatigue, nausea, and vomiting lasting for 2 days, without the typical diabetic symptoms of polydipsia, polyuria, or polyphagia. Laboratory tests revealed severe hyperglycemia (immediate serum glucose level, 29.7 mmol/L) and a glycated hemoglobin (HbA1c) value of 6.4%. Urine samples showed high levels of glucose (4+) and ketones (3+). Arterial blood gases indicated primary metabolic acidosis, with a pH of 7.29, a bicarbonate level of 12.5 mmol/L, and a lactate level of 1.5 mmol/L. Upon admission, the patient had a temperature of 37.2°C, blood pressure of 116/75 mmHg, heart rate of 101 beats a minute, respiratory rate of 20 a minute, and a 98% oxygen saturation (resting, room air); the BMI was 24.03 kg/m^2^. Physical examination revealed drowsiness, dry skin, and clinical dehydration; however, the results of other systemic examinations were negative. The other laboratory findings are shown in **Table 1**.

**Table 1:** Clinical characteristics of the patient during the two hospitalization events.

Variables	First hospitalization 19/11/2021	Second hospitalization 26/12/2021	Reference
random glucose (mmol/L)	29.7	32.4	
gas pH	7.29	7.18	7.35–7.45
PaCO_2_ (mmHg)	26	15	35.0–45.0
PaO_2_ (mmHg)	84		80.0–100.0
HCO3^-^(mmol/L)	12.5	5.6	21–27
BE (mmol/L)	-14.1	-22.8	-3–3
Lactate (mmol/L)	2.8	2.4	0.5–2.2
Potassium(mmol/L)	5.1	5.2	3.4–4.5
Sodium(mmol/L)	131	126	136–145
Urine glucose	4+	2+	–
Urine ketones	3+	3+	–
HbA1c (%)	6.4	9.4	
C-peptide(ng/ml)	0.1	0.1	1.1–4.4
Anti-GAD	–	–	
Anti-ICA	–	–	
Anti-Insulin	–	–	

We suspected newly developed diabetes mellitus coupled with ketoacidosis; hence, the patient was referred to the endocrinology department. Intravenous fluids to restore of circulatory volume and tissue perfusion, continuous insulin infusion treatment and correction of electrolyte imbalance were initiated immediately. After 1 day, by above active treatment, glucose gradually dropped below 13.9 mmol/L, urinary ketone turned negative, DKA resolved. Therefore, her insulin therapy was switched to subcutaneous insulin. Low levels of insulin (0.6 mIU/L) and C-peptide (0.1 ng/mL) indicated insulinogenic diabetes. The patient tested negative for typical type 1 diabetes mellitus (T1DM) autoantibodies including glutamic acid decarboxylase antibodies (anti-GAD), insulin autoantibodies (anti-insulin), and anti-islet cell antibodies (anti-ICA). Notably, the patient denied any history of diabetes or autoimmune disease. Other than sintilimab, no other potential causes of hyperglycemia (such as infection, pancreatitis, autoimmune diseases, Cushing’s syndrome, or drug exposure) were identified. The Naranjo Adverse Drug Reaction Probability Assessment Scale scored to 7 which suggests probable relation between the sintilimab and its reaction in patient. Therefore, we considered the possibility of ICI-DM, and discontinuing sintilimab immunotherapies. The patient was discharged with a prescription for multiple daily injections (insulin glargine at 8 units per day and insulin lispro at 6–8 units pre-meal).

**Figure 1:**
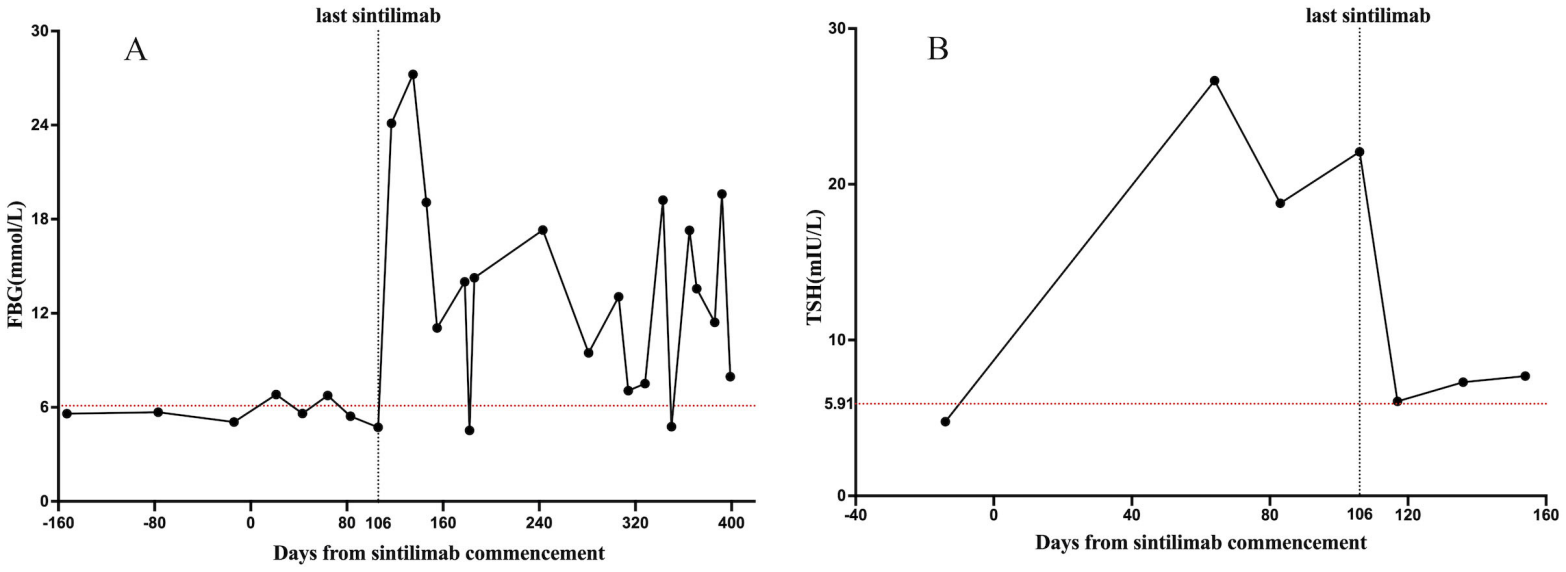
Changes in endocrine functions during sintilimab treatment. The X-axis shows the time interval since the start of the sintilimab treatment in days. The Y-axis shows the laboratory values of FBG (mmol/L) and TSH (mIU/L). **(A)** shows the change in FBG levels; **(B)** shows the change in TSH levels. The red dotted line is the upper limit for FBG and TSH. FBG, fasting blood glucose. TSH, thyroid-stimulating hormone.

However, 3 days after discontinuing insulin treatment, the patient returned to our hospital with life-threatening DKA. Blood test results revealed severe DKA (hyperglycemia, 32.4 mmol/L, acidosis pH 7.18; serum venous bicarbonate, 5.6 mmol/L) and an HbA1c level of 9.4%. The C-peptide values remained low. Upon resolution of DKA, the patient was discharged and subcutaneous insulin was continued to manage glycemia. Over the following year, the patient was closely followed at the outpatient clinic for insulin titration; nevertheless, the patient continued to show significant glucose fluctuations, similar to those in T1DM (**Figure 1A**).

ICI treatment can lead to multiple co-occurring irAEs. An elevated serum thyroid-stimulating hormone (TSH) level of 26.65 mIU/mL was observed 2 months before the onset of DKA, but it did not receive attention. Thyroid function tests revealed high TSH, normal free thyroxine (FT4), and negative thyroid peroxidase and thyroglobulin antibodies, indicating that the irAEs involved the hypothalamic-pituitary-thyroid axis. Therefore, a comprehensive assessment of the patient’s endocrine system was performed. Cortisol and adrenocorticotropic hormone (ACTH) levels were within the reference range, and no symptoms of adrenal insufficiency were reported. The relationship between TSH levels and sintilimab administration is shown in **Figure 1B**.

**Figure 2:**
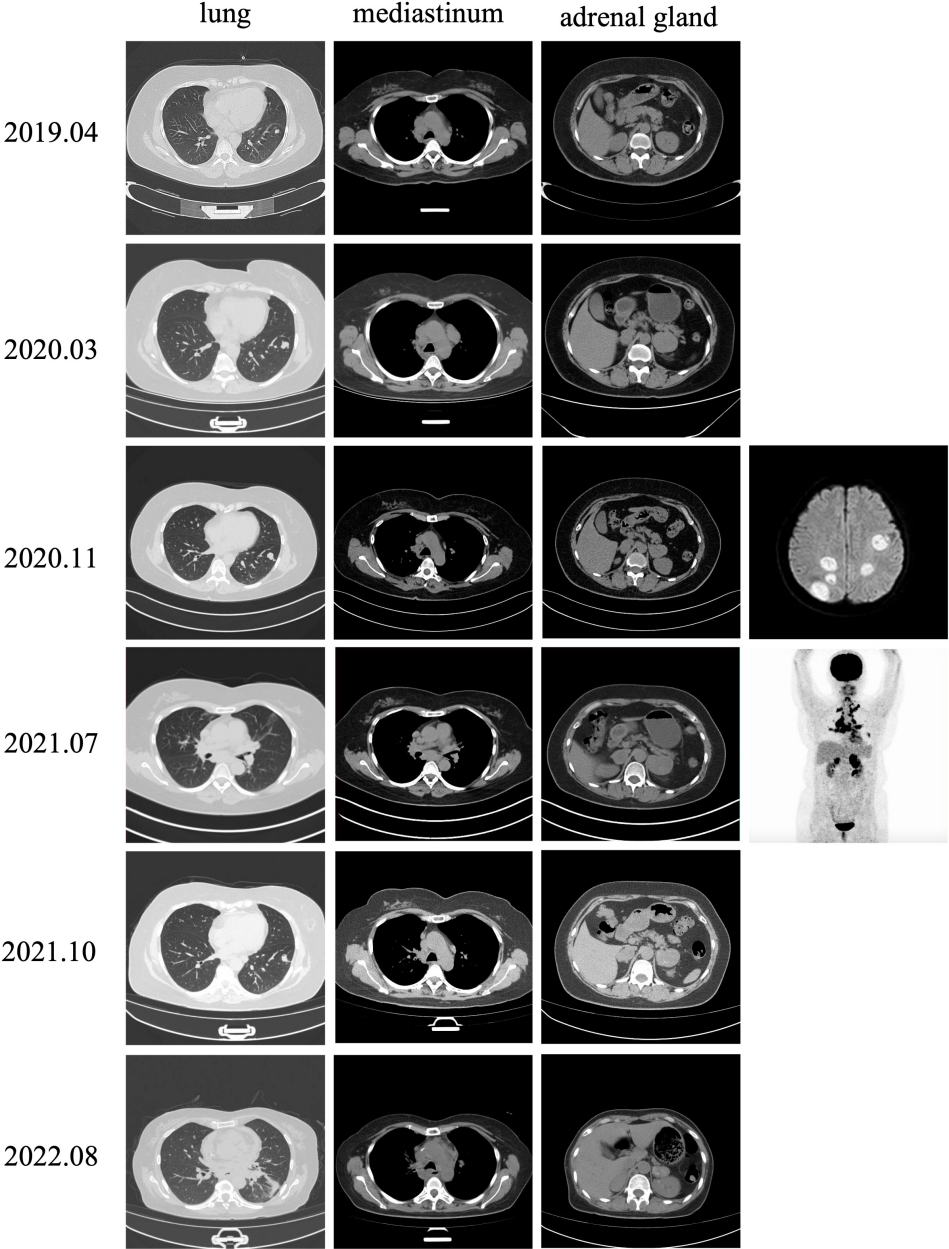
Image of disease progression.

**Figure 3:**
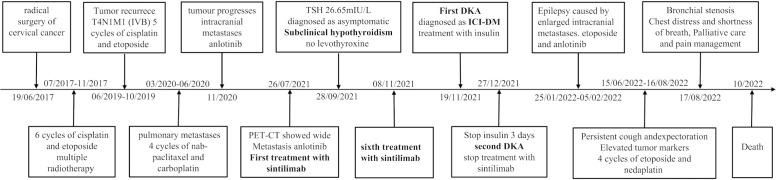
Timeline of disease progression.

The patient developed progressive dyspnea on August 17, 2022. Chest CT scans revealed enlarged lymph nodes compressing the principal bronchus (**Figure 2**), and bronchoscopy showed bilateral principal bronchus compression of approximately 50%, with a left lower lobar bronchus stenosis-like chink; therefore, bronchus stenting was performed. After consulting with the palliative team, the patient and her family discontinued treatment, and she transitioned to home hospice care. Unfortunately, the patient died in October 2022. The timeline for treatment is shown in **Figure 3**.

## 3 Discussion

IrAEs are systemic autoimmune diseases affecting any system through nonspecific T-cell activation. Non-endocrine irAEs are mostly associated with acute inflammation that can be resolved with glucocorticoid therapy, ultimately restoring organ function. IrEEs typically lead to chronic conditions and are frequently irreversible, requiring lifelong hormone replacement that, given ICI-induced immune activation, results in the destruction of most or all the hormone-producing cells; high-dose glucocorticoids are unlikely to reverse the damage and salvage the endocrine function of the gland. Glucocorticoids are not recommended for the treatment of ICI-DM, high doses of glucocorticoids can also increase glycemia (5). Due to clinical and pathophysiological similarities, the differential diagnosis between irEEs and primary autoimmune diseases may be challenging. IrEEs triggered by the different ICI treatments were different. Anti-cytotoxic T-lymphocyte antigen-4 (CTLA-4) agents are associated with the risk of adrenal insufficiency and hypophysis, whereas anti-programmed death-1 (PD-1)/programmed death ligand-1 (PD-L1) therapy is closely associated with insulin-dependent DM and thyroid dysfunction. Combination therapies are more toxic than ICIs. The onset time of irEEs varies among different ICI treatments. Anti-CTLA-4 therapy is usually administered within 12 weeks; however, anti-PD-1 therapy can be administered at any time between 0 and 48 weeks. ICI-DM can occur after 48 weeks and up to 2 years after initiating anti-PD-1 therapy. Therefore, the onset of ICI-DM is difficult to anticipate (6). In general, irEEs are strongly associated with high response rates and progression-free survival (7). According to the American Society of Clinical Oncology (ASCO) guidelines (8), ICIs should be discontinued, and patients should be managed for their effects until they improve if they have grade 2 adverse effects. IrEEs are distinct from other irAEs because of the typically irreversible hormonal deficits they cause, except for certain thyroid changes. The mainstay of treatment of irEEs is hormone replacement therapy. Except for endocrinopathies that improve with hormone replacement therapy, grade 4 irAEs generally require permanent discontinuation of ICIs.

ICI-DM is a comparatively rare irAE with a prevalence of 0.2–1.4% (9). ICI-DM is considered to be the result of autoimmune destruction of pancreatic β-cells. Recovery from autoimmune diabetes mellitus after the termination of ICIs treatment is not possible since the majority of β cells would be permanently damaged. Compared to classic TIDM, ICI-DM exhibits a rapid and fulminant onset of diabetes, characterized by the abrupt development of extreme hyperglycemia that the HbA1C is often elevated to 7.6%–9.7% at diagnosis so screening for HbA1c is not reliable in the patients (10). C-peptide levels remain low or absent in ICI-DM; however, 93% of patients with T1DM still showed detectable C-peptide levels 2 years after initial diagnosis (11). ICI-DM cases show obvious insulin deficiency and require lifelong insulin therapy; 60–85% of ICI-DM cases present with DKA (12, 13). One-third of patients with ICI-DM show elevated serum amylase and lipase, suggesting that exocrine pancreatic inflammation may also play a role in the pathogenesis (14). The level of autoantibodies in patients with T1DM is markedly lower than that in patients with classic T1DM (40–50% vs 90%), and patients with positive autoantibodies for ICI-DM have an earlier onset of diabetes and a higher risk of DKA (15). As in T1DM, genetic factors such as HLA typing correlate with the risks of developing ICI-DM, especially HLA-DR4 (16). Collectively, these studies indicate that the pathophysiology of ICI-DM is unique and distinct from that of classic T1DM.

Several treatments are effective in preventing ICI-DM in NOD mice, including JAK1/2 inhibitors, anti-CD3, anti-TNF-a, and anti-IFN-γ. However, none of these have been successful in treating humans with confirmed ICI-DM. Although two patients with ICI-DM were insulin-free after infliximab therapy, it was difficult to determine the cause of the hyperglycemia, as neither had overt insulin deficiency, such as insufficient C-peptide or obvious DKA (15). In a retrospective study of 735 patients with ICI-DM diagnosed between 2015 and 2019, the incidence increased since 2015. In patients with ICI-DM, 24.90% presented with fulminant-type 1 diabetes, whereas 45.99% had diabetic ketosis or DKA. Approximately 25% of the patients with ICI-DM show life-threatening or fatal outcomes (17). In our study, the patient not only developed ICI-DM (grade 4) after receiving sintilimab but also experienced thyroid dysfunction (grade 2). Sudden onset of DKA is the first manifestation, and the patient showed nearly undetectable levels of insulin, low C-peptide, negative islet-cell autoantibodies, and a slightly elevated HbA1c level of 6.4% at diagnosis. Acute and rapid islet dysfunction commonly occurs in patients with fulminant-type 1 diabetes mellitus. Three days after discontinuing insulin treatment, the patient returned to our hospital with life-threatening DKA.

For irEEs, except for hypophysitis with compression of the optic chiasm or optic nerve or severe thyroid eye disease, there is no need to interrupt or stop ICI treatment. Unfortunately, sintilimab treatment was discontinued because of the severe symptoms of DKA. In fact, enhanced clinical awareness of abnormal glucose levels in patients before the diagnosis of CIADM may lead to early identification of ICI-DM and prevention of potential DKA. ICI-DM is a relatively rare but potentially life-threatening adverse reaction to ICI treatment. Clinicians must take this seriously because of its high incidence, serious consequences of missed diagnosis, the need for lifelong continuous insulin treatment, related risk of diabetes mellitus complications, and reduced survival (15). The ASCO recommends monitoring random blood glucose at baseline and every cycle of ICI therapy, particularly in patients treated with anti-PD-1. In cases of new-onset hyperglycemia, the basal metabolic profile, blood pH, serum and urine ketone, HbA1c, and C-peptide levels should be determined. These assays can be used to identify patients with DKA and differentiate between different types of hyperglycemia such as stress hyperglycemia, steroid-induced hyperglycemia, and type 2 diabetes mellitus (T2DM) (8). Once DKA is confirmed, hospitalization is indicated, and a standard typical should be immediately administered, including fluid resuscitation, continuous intravenous insulin infusions, and correction of electrolyte abnormalities. In severe cases, monitoring in the intensive care unit is mandatory. It is essential to treat any correctable underlying cause of DKA, the most common incentives is infection. Evaluation of insulin, C-peptide, and insulin antibodies is recommended at ICI-DM diagnosis. Co-management with an endocrinologist may help optimize insulin doses that contribute to better glycemic control and HbA1c. Patient education is also fundamental, particularly regarding glucose monitoring and symptom management after discharge. Patients should be instructed to promptly report any symptoms of diabetes, such as acute polyuria, polydipsia, and weight loss, and seek immediate evaluation, especially in the presence of fever and/or infection, even if their symptoms are not severe.

The thyroid gland is the most commonly affected endocrine organ (30%) (5). The median time for thyroid dysfunction to appear after ICI treatment is 6 weeks. The risk factors of ICI-induced thyroid dysfunction include females, high BMI, increased 18F-deoxyglucose uptake in the thyroid, high TSH, and thyroid autoantibodies (10). Patients are often diagnosed based on abnormal thyroid function rather than symptoms, although they may present with symptoms of hypothyroidism, such as anorexia, fatigue, weight gain, and constipation. Notably, these symptoms are not always present (18). Thyroid dysfunction during PD-1 inhibitor treatment correlates with improved responses and can be used as a predictive factor for improved treatment outcomes (19). During the 9-week sintilimab treatment, our patient developed thyroid dysfunction and was diagnosed with asymptomatic subclinical hypothyroidism. In addition to sintilimab therapy, the patient received anlotinib, a tyrosine kinase inhibitor (TKI). In a single-center retrospective study of 126 patients who received PD-1 inhibitor therapy, 23% experienced thyroid dysfunction. TKIs were identified as significant triggers of hypothyroidism in 63.2% of the patients (20). After 2 weeks of sintilimab discontinuation and continued use of arotinib, her thyroid function returned to normal without levothyroxine. Therefore, we considered sintilimab to be the prime predisposing factor for thyroid dysfunction.

The TSH level is the most sensitive and preferred biochemical test for diagnosing thyroid dysfunction in patients. Thyroid function should be assessed at least every 4–8 weeks in patients receiving ICIs to monitor for potential thyroid dysfunction. Thyroid hormone supplementation was administered to symptomatic patients with elevated TSH levels or asymptomatic patients with TSH levels >10 mIU/mL. Levothyroxine can be initiated at a daily dose of 25–50 mg, and the dosage must be adjusted based on serum TSH levels (8). Brain imaging and pituitary hormone tests should be conducted to identify central hypothyroidism if T4 and TSH levels are low (21).

Many questions about irAEs remain unanswered. These include identifying risk factors and biomarkers with predictive value, predicting the onset and severity of adverse events, and further demonstrating the relationship between irEEs and clinical outcomes. This could enable clinicians to stratify patients according to the risk and take the necessary steps to manage patients with iris to avoid permanent discontinuation of ICI therapies, especially in highly effective anti-tumor responses. In addition to retrospective studies, prospective studies are needed. IrEEs present distinct clinical challenges. Providing medical counseling to patients and their families about the possibility of irEEs before initiating ICI therapy increases awareness of potential adverse events, improves coping abilities, raises medical compliance, and improves clinical outcomes. Non-endocrinologists should identify endocrine dysfunction in patients with nonspecific symptoms or complex abnormal laboratory results. Comprehensive patient education will help in the early diagnosis of endocrine disorders. Effective cooperation between the endocrinology and oncology departments can enhance the prospects of patients with irEEs.

## Data Availability

The original contributions presented in the study are included in the article/supplementary material, further inquiries can be directed to the corresponding author/s.
